# Chemical Potential Analysis as an Alternative to the van’t Hoff Method: Hypothetical Limits of Solar Thermochemical Hydrogen

**DOI:** 10.1021/jacs.4c02688

**Published:** 2024-05-13

**Authors:** Stephan Lany

**Affiliations:** National Renewable Energy Laboratory, Golden, Colorado 80401, United States

## Abstract

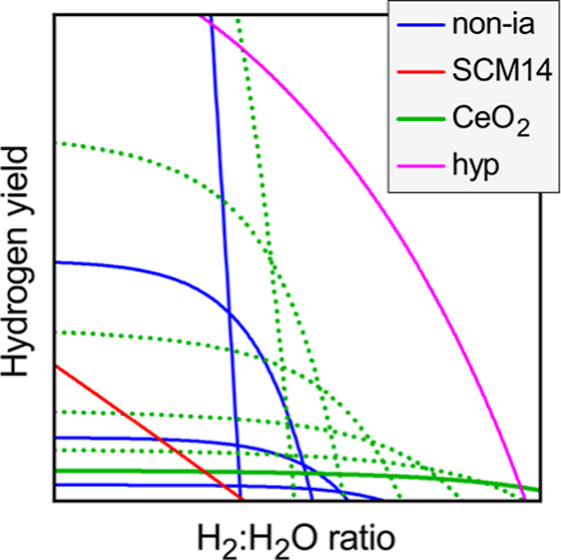

The van’t Hoff method is a standard approach for determining reaction enthalpies and entropies, e.g., in the thermochemical reduction of oxides, which is an important process for solar thermochemical fuels and numerous other applications. However, by analyzing the oxygen partial pressure *p*O_2_, e.g., as measured by thermogravimetric analysis (TGA), this method convolutes the properties of the probe gas with the solid-state properties of the examined oxides, which define their suitability for specific applications. The “chemical potential method” is here proposed as an alternative. Using the oxygen chemical potential Δμ_O_ instead of *p*O_2_ for the analysis, this method does not only decouple gas-phase and solid-state contributions but also affords a simple and transparent approach to extracting the temperature dependence of the reduction enthalpy and entropy, which carries important information about the defect mechanism. For demonstration of the approach, this work considers three model systems; (1) a generic oxide with noninteracting, charge-neutral oxygen vacancy defects, (2) Sr_0.86_Ce_0.14_MnO_3(1−δ)_ alloys with interacting vacancies, and (3) a model for charged vacancy formation in CeO_2_, which reproduces the extensive experimental TGA data available in the literature. The reduction behavior of these model systems obtained from the chemical potential method is correlated with simulated results for the thermochemical water splitting cycle, highlighting the exceptional behavior of CeO_2_, which originates from defect ionization. The theoretical performance limits for solar thermochemical hydrogen within the charged defect mechanism are assessed by considering hypothetical materials described by a variation of the CeO_2_ model parameters within a plausible range.

## 1 Introduction

Solar thermochemical splitting of H_2_O and CO_2_ is one of the few potential routes toward direct solar-to-fuel conversion on an industrial scale,^1^ providing a perspective for renewable energy beyond electricity-based sources and a path to synthetic alternatives for petroleum-derived liquid hydrocarbon fuels.^2^ However, this technology is currently still held back by the lack of oxide working materials with optimal thermodynamic properties for the two-step reduction–oxidation cycle.^1^ While ceria (CeO_2_) is recognized as the current benchmark system,^3,4^ it requires undesirably high temperatures to achieve sufficient O deficiency in the reduction step, which has motivated both experimental and computational material design and discovery efforts aimed at either modifying CeO_2_^5,6^ or identifying suitable new oxides.^4,7−15^ However, any gains in improved reduction behavior are offset by deterioration of the oxidation behavior, often dramatically limiting the H_2_ generation during the water splitting step to very dilute H_2_/H_2_O ratios well below 1:100,^7,8,16^ whereas CeO_2_ continues to split water even above 1:10. Materials design is often guided by a theoretical performance limit described as a function of specific material properties, such as, in photovoltaics, the Shockley–Queisser limit and extensions thereof.^17−19^ For a defect-mediated solar thermochemical hydrogen (STCH) cycle, for example, current design principles provide little more than the expectation that suitable values of O vacancy formation energies lie in the fairly wide range between 2 and 5 eV.^12,20,21^

Complicating the material design is the fact that the viability and performance of STCH oxides can depend on numerous material features beyond the formation energy of an isolated, charge-neutral O vacancy. From a computational perspective, the defect equilibrium can be strongly affected by defect interactions, compositional complexity and disorder, and by excitation of free (polaron- or band-) electrons.^6,22−27^ Within this realm of possibilities, it is currently unclear where the “sweet spot” between the reduction and oxidation properties lies and how it can be realized in real materials. Experimentally, the thermochemical redox behavior is characterized through van’t Hoff analysis of reduction enthalpies and entropies. The fact that the exceptionally large entropy observed in ceria^28,29^ can be related to its ability to sustain high H_2_/H_2_O ratios^23^ illustrates that the performance optimization via material design can greatly benefit from feedback between computational model predictions and experimental thermochemical analysis, provided the establishment of a “common language”. The present work suggests that the traditional van’t Hoff method does not serve this purpose well because it conflates solid-state and gas-phase contributions and because it is ill-suited to resolve the temperature dependence of enthalpy and entropy, which contains important information. The following sections will describe the alternative “chemical potential” method and illustrate its utility on the basis of simulated data for three model systems, showing that the temperature dependence of enthalpy and entropy reveals important insights about the defect mechanism. This information remains obscured in the traditional van’t Hoff method but provides valuable guidance for material design. The final section will analyze the impact of the different defect mechanisms and their material parameters on the water splitting performance, expressed by the H_2_ yield per cycle and the H_2_/H_2_O ratio in the oxidation step.

## 2 Results and Discussion

### 2.1 Chemical Potential Analysis of Reduction Enthalpy and Entropy

This paragraph briefly reviews the relations between thermodynamic variables during the thermochemical reduction of an oxide, broadly reflecting the terminology used in the preceding literature, e.g., refs (23, 30, and 31). For simplicity and generality, the stoichiometric ratios in the reaction
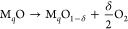
1are normalized such that the fully oxidized form has an O content of unity with a fractional coefficient *q* for the metal (M) cation(s). At temperature *T*, the total free energy gain associated with the reduction reaction, eq 1, is
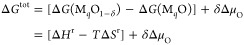
2where Δ*G*^r^ = Δ*H*^r^ – *T*Δ*S*^r^ represents the solid-state contributions to free energy, enthalpy, and entropy, in terms of absolute energies per normalized formula unit (nfu), as defined in the reduction reaction eq 1. The O chemical potential  is measured relative to the 0 K elemental reference state of the O_2_ molecule. Using  at the equilibrium O deficiency δ, we obtain the relation between Δμ_O_ and the differential reduction enthalpy and entropy per O atom
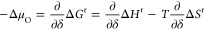
3where we will use the shorthand notations δ*G*^r^, δ*H*^r^, and δ*S*^r^ for the differential quantities for the remainder of this work. From eq 3, it further follows that .^23^

Within the ideal gas law, the gas-phase chemical potential at a given temperature *T* is related to the respective partial pressure *p* by (see, e.g., refs (27, 32, and 33))
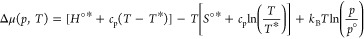
4where standard pressure *p*° = 1 bar at T* = 298.15 K with the respective enthalpy *H*^°^* and entropy *S*^°^* serves as a reference condition. Neglecting vibrational contributions, the constant-pressure heat capacity *c*_p_ is a constant, e.g., *c*_p_ = 3.5*k*_B_ = 29.1 J/mol/K for diatomic molecules, where *k*_B_ is the Boltzmann constant. Using *H*^°^* = 8.7 kJ/mol and *S*^°^* = 205.1 J/mol/K for O_2_,^34^eq 4 agrees with tabulated free energies at 1 bar^35^ to within 35 meV/O up to 2000 K, illustrating the accuracy of this expression within the range of (*p*,*T*) conditions of interest (see also additional discussion of vibrational effects in the Supporting Information and correction formula for inclusion of vibrational contributions in the repository). Rearranging eq 4 and inserting eq 3, we obtain
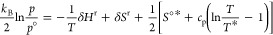
5where the simplification used the fact that the nonideality of the standard enthalpy is negligible, i.e., *H*^°^* – *c*_p_T* < 0.1 meV/O_2_. Equation 5 represents the linear form of the van’t Hoff equation but reveals the convolution of the solid-state enthalpy and entropy contributions with gas-phase contributions (square-brackets), including the residual temperature-dependent term.

**Figure 1:**
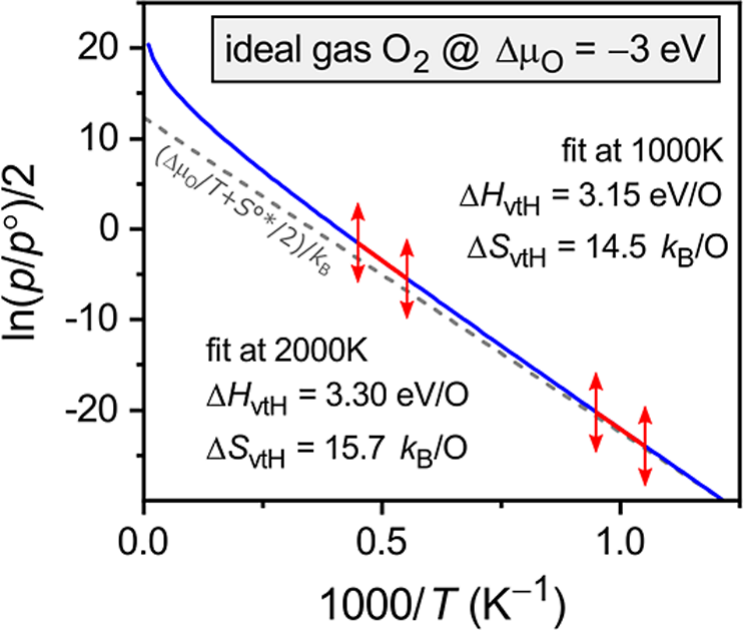
van’t Hoff plot of the O_2_ gas-phase partial pressure as a function of 1/*T* for the case of a constant O chemical potential Δμ_O_ = −3 eV (δ*H*^r^ = 3 eV, δ*S*^r^ = 0) in the temperature range *T* > 800 K (blue line). Enthalpies Δ*H*_vtH_ and entropies Δ*S*_vtH_ are obtained by fits around 1000 and 2000 K using traditional van’t Hoff analysis. Dashed gray line indicates the idealized linear relationship corresponding to Δ*H*_vtH_ = −Δμ_O_ = 3.0 eV/O and Δ*S*_vtH_ = *S*^°^*/2 = 12.34 *k*_B_/O.

Traditional van’t Hoff analysis usually does not attempt to quantitatively separate solid-state and gas-phase contributions,^4,10,16,36−39^ although there are a few exceptions.^40^ Instead, fitting experimental results to
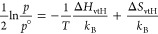
6yields Δ*H*_vtH_ (negative of the slope) and Δ*S*_vtH_ (intercept), which are taken as the material characteristics of the reduction behavior. Even when it is acknowledged that the gas-phase contribution accounts for about 15 *k*_B_/O = 125 J/mol/K of the entropy^29,39,41^ at the temperatures of interest, its temperature dependence is usually neglected. To illustrate the effect of the gas-phase contributions, Figure 1 shows the van’t Hoff plot, eq 5, for a constant chemical potential at reducing conditions, Δμ_O_ = −3 eV (e.g., approximately *p*O_2_ = 10^–5^ bar at *T* = 1500 °C). In the case of a constant temperature-independent O chemical potential, we have δ*H*^r^ = −Δμ_O_ for the reduction enthalpy and δ*S*^r^ = 0 for the entropy (cf. eq 3). Figure 1 also shows two fits of the form used in traditional van’t Hoff analysis, eq 6, one around 1000 K and one around 2000 K, roughly corresponding to the typical range of experimental temperatures in thermogravimetric analysis (TGA). We observe that the entropy Δ*S*_vtH_ varies by as much as 1.2 *k*_B_/O between 14.5 and 15.7 *k*_B_/O over this temperature range. While this variation appears relatively small compared to the total gas-phase contribution, its magnitude is comparable to the solid-state entropies, which are the actual material properties of interest. As we will see in Section 2.2 for specific model systems, the entropies δ*S*^r^ are often only in the range of a few *k*_B_/O, especially at higher defect concentrations and in the presence of repulsive defect interactions.^27^ Therefore, the variation observed in Figure 1 is clearly undesirable. Similarly, the enthalpy Δ*H*_vtH_ deviates by up to 0.3 eV/O from the “true” reduction enthalpy δ*H*^r^ = 3.0 eV, which is too significant to ignore and which hampers the comparison of experimental data with first-principles calculations.

A simple approach for obtaining more precise estimates of the solid-state reduction enthalpies δ*H*^r^ and entropies δ*S*^r^ from a van’t Hoff type analysis is to subtract the gas-phase term from either side of eq 5
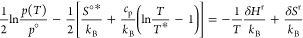
7

Using this form in lieu of eq 6 affords direct fitting of δ*H*^r^ and δ*S*^r^ without the gas-phase contributions. Alternatively, I propose the “chemical potential method”, i.e., converting the partial pressures into chemical potentials via eq 4 (considering that ) and utilizing eq 3, i.e.,
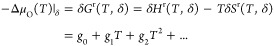
8where *g*_*i*_ are polynomial expansion coefficients. Within a linear model, eq 8 is equivalent to eq 7 and yields δ*H*^r^ = *g*_0_ from the intercept and δ*S*^r^ = −*g*_1_ from the slope of δ*G*^r^(*T*) at a constant value of δ. However, the advantage of eq 8 is that the extension to higher orders in *T* (or to other functional forms) is straightforward, thereby providing a simple and transparent approach for determining the temperature dependence of the reduction enthalpy and entropy, i.e., by using the relationships
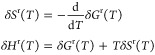
9

### 2.2 Exemplification by Model Systems

So far, no specific reduction mechanism has been invoked. For example, the reduction could occur by the formation of O vacancy defects or by a phase transition (in the latter case, M_*q*_O_1−δ_ could represent a phase mixture between two ordered oxides with an oxidized and a reduced stoichiometry). In the following, we will discuss three different O vacancy (*V*_O_) defect mechanisms, each represented by a model system: (1) a generic oxide M_*q*_O_1−δ_ with noninteracting (non-ia), charge-neutral O vacancies, exemplified by a system with a defect formation energy of Δ*H*_D_^ref^ = 2.5 eV at μ_O_ = Δμ_O_^ref^ (Δμ_O_ = 0). (2) A system with interacting, charge neutral vacancies. We recently developed such a model for Sr_1–*x*_Ce_*x*_MnO_3(1−δ)_ alloys on the basis of the concept of the free energy of defect interaction Δ*G*_D_^int^(*T*).^27^ For the present purpose, we will consider a Ce composition of *x* = 0.14 (SCM14 perovskite). Including the small adjustment (−0.18 eV) of the first-principles calculated dilute-limit formation energy of *V*_O_ in SrMnO_3_,^27^ this model yields very close agreement with the TGA data for SCM14 in ref (16) over the full range of experimental (*p*O_2_, *T*) conditions. (3) As a benchmark material for thermochemical reduction, CeO_2_ has an extensive body of literature data,^28,29,42−44^ which has been combined by Zinkevich et al.^45^ into a phenomenological thermodynamic model. Section 2.3 below describes a charged vacancy defect mechanism for CeO_2_, which, again with only minor adjustments to first-principles results, reproduces very closely the model of ref (45). (Note that the term “charged defect mechanism” should be understood to include the ionization process, but not necessarily to imply complete ionization under all conditions.) The defect model and computational simulation of (*p*O_2_, *T*, and δ) data will be discussed in detail below in Section 2.3, but for now we will use this data as a stand-in for experiments to conclude the discussion of the different analysis methods.

**Figure 2:**
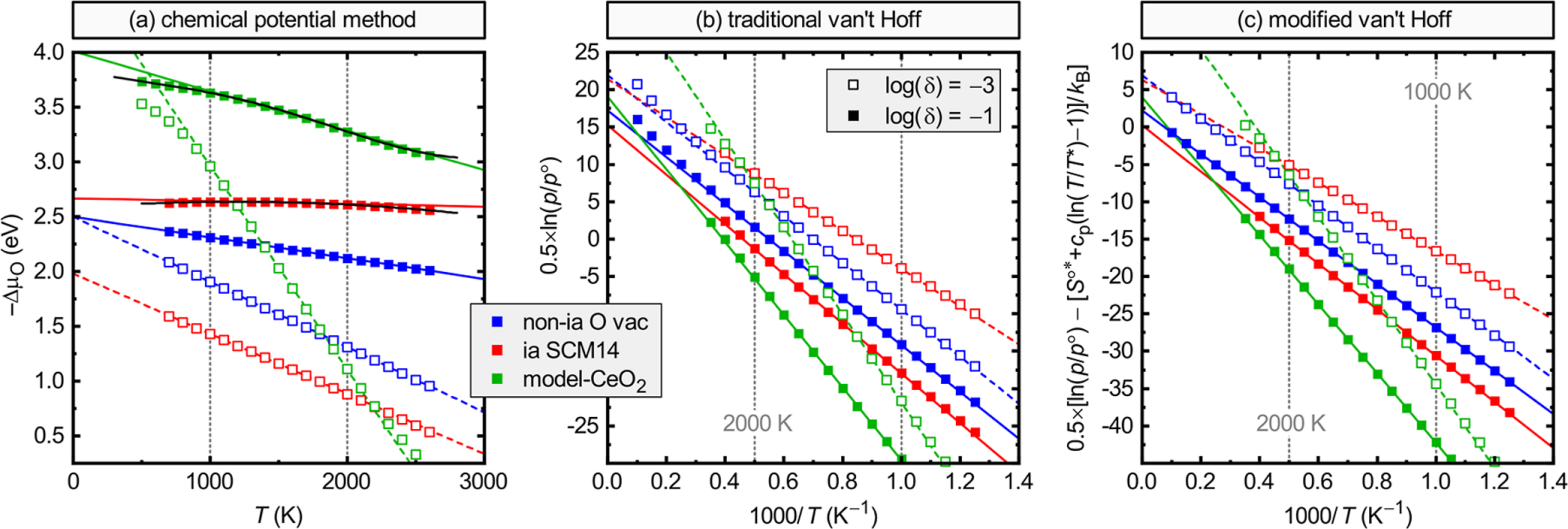
Analysis of reduction enthalpies and entropies, comparing the (a) chemical potential, (b) traditional van’t Hoff, and (c) modified van’t Hoff methods. Three model systems are considered: (blue) a generic oxide M*_q_*O_1−δ_ with noninteracting, charge-neutral *V*_O_^0^ vacancies with a formation energy of Δ*H*_D_^ref^ = 2.5 eV. (red) Sr_0.84_Ce_0.14_MnO_3(1−δ)_ (SCM14) as described by the model of ref (27) for interacting (ia) defects. (green) CeO_2(1−δ)_ as described by the charged *V*_O_^+2^ model of the present work. For each system, the fitting was performed for δ = 0.001 and 0.1 over the temperature interval 1000 K ≤ *T* ≤ 2000 K. (a) also includes a 2nd order fit for SCM14 and a 5th order fit for CeO_2_ (black).

For the three model systems, Figure 2 compares the thermochemical analysis in the proposed chemical potential method (eq 8) with the traditional (eq 6) and modified (eq 7) van’t Hoff methods. Here, we consider two stoichiometries, δ = 0.001 and 0.1, roughly corresponding to the range accessible in TGA experiments.^16,29^ The respective fit results for δ*H*^r^ and δ*S*^r^ are given in Table 1. Note that the Table gives only linear fit results for the chemical potential method; higher order fit results (cf. black curves in Figure 2a), which provide information about the temperature dependence of enthalpy and entropy, are discussed below in Section 2.4.

**Table 1:** Thermochemical Parameters δ*H*^r^ and δ*S*^r^ Obtained from the Linear Fits (1000 K ≤ *T* ≤ 2000 K) Shown in Figure 2 for the Three Model Systems, Using the Three Methods Represented by Equation 8 (chem-pot), Equation 6 (vtH-trad), and Equation 7 (vtH-mod)^a^

model	analysis	δ	δ*H*^r^ (eV)	δ*S*^r^ (*k*_B_)	δ*H*^r^ (kJ/mol)	δ*S*^r^ (J/mol/K)
non-ia *V*_O_^0^	chem-pot	0.001	2.500	6.91	241.2	57.4
	vtH-mod	0.001	2.500	6.91	241.2	57.4
	vtH-trad	0.001	2.707	21.95	261.2	182.5
	chem-pot	0.1	2.500	2.20	241.2	18.3
	vtH-mod	0.1	2.500	2.20	241.2	18.3
	vtH-trad	0.1	2.707	17.24	261.2	143.4
ia SCM14	chem-pot	0.001	1.981	6.37	191.1	53.0
	vtH-mod	0.001	1.977	6.34	190.8	52.7
	vtH-trad	0.001	2.184	21.39	210.7	177.8
	chem-pot	0.1	2.666	0.29	257.2	2.4
	vtH-mod	0.1	2.658	0.22	256.4	1.8
	vtH-trad	0.1	2.864	15.26	276.4	126.9
CeO_2_*V*O^+2^	chem-pot	0.001	4.827	21.58	465.8	179.5
	vtH-mod	0.001	4.828	21.61	465.8	179.7
	vtH-trad	0.001	5.034	36.65	485.7	304.8
	chem-pot	0.1	4.008	4.19	386.7	34.8
	vtH-mod	0.1	3.986	4.01	384.6	33.4
	vtH-trad	0.1	4.193	19.06	404.6	158.5

a The enthalpies and entropies are given both in microscopic (eV, *k*_B_) and macroscopic (kJ/mol, J/mol/K) units, where mol refers to the number of O atoms.

For the noninteracting charge-neutral defect model, the chemical potential method, Figure 2a, exactly reproduces the model parameters, i.e., δ*H*^r^ = Δ*H*_D_^ref^ = 2.5 eV for the reduction enthalpy for both values of δ, and  for the (differential) reduction entropy of an ideal solution (see Section 2.3), i.e., a random distribution of O atoms and vacancies (note that the neutral defect model does not include the mixing entropy of electron-polarons because it would require vacancy-electron dissociation, i.e., a charged defect mechanism, as discussed in more detail in Section 2.3). The traditional van’t Hoff method exhibits significant deviations from the ideal linearity, which, on the scale of the plot in Figure 2b, become apparent only at high temperatures above 2000 K but nevertheless affect the numerical analysis. It yields Δ*H*_vtH_ and Δ*S*_vtH_ with offsets of 0.21 eV and 15.05 *k*_B_, respectively, from the model that generated the data, reflecting the standard enthalpy and entropy of O_2_ in the temperature range used for the fit. These numbers are close to, although not exactly the same as, the respective values at the middle of the temperature interval (*H*° = 0.23 eV and *S*° = 15.16 *k*_B_ at 1500 K). Thus, subtraction of these values from a traditional van’t Hoff analysis, which is similar to the approach taken in ref (40), should give reasonably accurate values for the solid-state enthalpies and entropies. However, appropriate values need to be reevaluated depending on the respective experimental temperature range. In contrast, the modified van’t Hoff method, Figure 2c, restores the exact linearity for the noninteracting defect case and yields the same results as the chemical potential method.

In the case of the interacting defects in SCM14, we now observe that the reduction enthalpy δ*H*^r^ increases considerably with the defect concentration δ, reflecting the enthalpy penalty due to repulsive defect interactions. At the same time, the entropy δ*S*^r^ is reduced relative to the ideal solution behavior of noninteracting defects because the interacting defect distribution is no longer random. At the higher concentration δ = 0.1, the reduction of the differential entropy is so strong that it almost eliminates the random mixing entropy, δ*S*^r^ = 0.3 *k*_B_ for SCM14 compared to 2.2 *k*_B_ for noninteracting defects (see Table 1). Note, however, that the absolute entropy, Δ*S*^r^ in eq 2 is much less affected (see ref (27) for a more detailed discussion). The effects of defect interactions, i.e., causing an entropy reduction and a concentration dependence of the enthalpy, are apparent in Figure 2a–c in all analysis methods, although the chemical potential method provides perhaps the most immediate reflection of these consequences. Further, we observe nonlinearity in the SCM14 data, which is especially pronounced at the higher defect concentration δ = 0.1, and which we will study in more detail in Section 2.4 below. In the traditional van’t Hoff method, Figure 2b, this nonlinearity is masked by the inherent convolution with the nonlinearity of the gas-phase contribution, and even in the modified van’t Hoff plot, Figure 2c, it is hardly discernible. Table 1 also reveals minor differences between the “chem-pot” and “vtH-mod” values for SCM14, reflecting differences in the root-mean-square minimization in cases where the linear relationship is not perfect.

The analysis of the model data for CeO_2_ yields large values for the reduction enthalpy, reaching up to δ*H*^r^ = 5.0 eV (486 kJ/mol) in the traditional van’t Hoff analysis, in agreement with well-established literature results.^29^ When considering the solid-state only enthalpies, δ*H*^r^ decreases with increasing defect concentrations, from 4.8 eV at the lower concentration δ = 0.001 to 4.0 eV at δ = 0.1 (Table 1), in contrast to the SCM14 interacting defect model. This concentration dependence has not been satisfactorily explained in the literature and raises the question of which of the values can be compared with defect formation energies from first-principles calculations. The change of δ*H*^r^ is accompanied by a corresponding reduction of the entropy from δ*S*^r^ = 21.6 to merely 4.2 *k*_B_. In the traditional van’t Hoff analysis, the magnitude of the effect is somewhat concealed by the gas-phase contribution (36.7 vs 19.1 *k*_B_, see Table 1). Notably, the solid-state entropy values in CeO_2_ values far exceed the respective ideal solution mixing entropies of the noninteracting model system (cf. Table 1), indicating a very different defect mechanism. The original model of charged *V*_O_^+2^ vacancies and Ce(3+)^−1^ small polaron carriers^29^ yields a value of 17.9 *k*_B_ at δ = 0.001 (see Supporting Information), comparable but noticeably lower than the measurement (about 20.5 *k*_B_ in ref (29) after subtraction of the gas-phase entropy). However, cluster-expansion Monte Carlo simulations suggested that short-range order effects on the Ce sublattice further reduce the entropy,^22^ which would imply a more significant discrepancy between model and measurement. Reference (46) suggests that the shortfall could be explained by a large on-site entropy contributions due to electronic excitations within Ce(3+) polarons. The present defect model of charged vacancies with delocalized band-electrons (Section 2.3) reproduces the experimental data well without on-site contributions, but it should be noted that it accounts for a similar effect because the conduction band continuum of CeO_2_ consists dominantly of the same Ce-4f states that form the Ce(3+) electron-polaron.

Given that CeO_2_ is the prototypical oxide for STCH, the observation of high reduction enthalpies up to 5 eV has certainly influenced the common belief that suitable defect formation energies Δ*H*_D_^ref^ for thermochemical hydrogen production lie in a range of about 2–5 eV.^12,20,21^ This wide range essentially reflects a corresponding wide range of associated reduction entropies and, therefore, can be narrowed down by making assumptions about δ*S*^r^.^47,48^ However, the exceptionally large entropies in CeO_2_ have so far not been replicated in other STCH oxides, and the upper end of the enthalpy range may be entirely unsuitable for STCH in systems that do not exhibit the high-entropy behavior of CeO_2_. Therefore, it is highly desirable to identify the signatures of ceria-like behavior in the thermochemical properties. We observe in Figure 2a clear nonlinear behavior for CeO_2_ for both compositions, indicating that there should be a discernible temperature dependence of the enthalpies and entropies. Thus, after the specification and validation of the defect model in the following section, we will turn toward a detailed analysis of the temperature and composition dependence δ*H*^r^(*T*, δ) and δ*S*^r^(*T*, δ) in Section 2.4, illustrating how such an analysis can reveal important insights about the underlying defect mechanism.

### 2.3 Computational Simulation of Different Defect Mechanisms

The equilibrium defect concentration is determined by minimization of Δ*G*^tot^ (eq 2) with respect to δ. Within a noninteracting, charge-neutral O vacancy defect mechanism with a single nonequivalent O site, we obtain
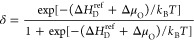
10where the absolute reduction enthalpy per formula unit equals the sum of the formation energies of all defects, Δ*H*^r^ = δΔ*H*_D_^ref^, and the absolute reduction entropy equals the ideal solution mixing entropy Δ*S*^r^ = −*k*_B_[δ ln(δ) + (1 – δ) ln(1 – δ)]. Thus, we have the simple analytical expressions δ*H*^r^ = Δ*H*_D_^ref^ and  for the corresponding differential quantities, which are exactly reproduced by the chemical potential analysis discussed above.

The idealized noninteracting defect model often becomes less accurate as the defect concentrations increase above a certain threshold, typically in the order of δ = 0.01, where repulsive defect interactions become important.^26,27,33^ In ref (27), we introduced a model for the free energy of defect interaction, which was parametrized as Δ*G*_D_^int^(δ,*x*,*T*) for Sr_1–*x*_Ce_*x*_MnO_3(1−δ)_ alloys. Within this model, the defect interactions result in an additive term δΔ*G*_D_^int^ in the total free energy Δ*G*^tot^ and corresponding contributions in the equilibrium concentrations (cf. eq 10). The results of the present work use the second order expansion of Δ*G*_D_^int^ in *T* for SCM14 with the Ce composition *x* = 0.14 (the parameters and simulation code are available in the Supporting Information of ref (27)).

Another effect that can dramatically change the thermochemical reduction behavior is defect ionization. By definition, the neutral vacancy mechanism, which includes the present SCM14 model, involves electrons that are tightly bound to the vacancy, often manifesting themselves in the form of a reduced oxidation state at two cation metal neighbor atoms. The additional entropy contribution from the distribution of the electron-polarons over the cation sublattice occurs only if the electrons dissociate from the vacancy, thereby creating ionized, charged vacancies. However, the entropy gain has to be weighed against the enthalpy cost, i.e., the ionization energy. To quantify this free energy contribution, band gap corrected hybrid functional calculations were performed for charged *V*_O_ defects and Mn(3+) polarons in perovskite SrMnO_3_, the base material for SCM alloys (for methods details, please see Section 4 and ref (27)). The vacancy transition levels are obtained at 0.72 and 0.88 eV below the conduction band minimum (CBM), whereas the polaron state is 0.10 eV below the CBM, resulting in a total ionization energy of 1.40 eV per defect. Using the expression for the polaron entropy (see Supporting Information), we find that charged vacancies become favorable only at low defect concentrations and high temperatures (δ ≤ 0.001 and *T* ≥ 1400 °C). However, under STCH relevant conditions (higher δ and/or lower *T*), the neutral mechanism dominates. This behavior is likely prevalent in many 3d transition metal oxides, where the lack of the electronic entropy contribution results in a strong limitation of the H_2_ partial pressure,^23^ as observed in water splitting experiments.^7,8,16^ However, the topic deserves further studies as the discovery of oxides with high reduction entropies could have a high impact on STCH.

For the charged defect mechanism, which requires more favorable defect ionization energies, we consider that the electrons can not only form dissociated polarons but also be excited into the continuum of conduction band states. Absent charges due to other types of defects, the equilibrium is now subject to a charge balance condition for the concentrations *n* of electron (e) and hole (h) carriers and of charged defects
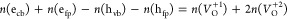
11Here, the left-hand side accounts for the net negative charge due to conduction-band (cb), valence-band (vb), and free polaron (fp) carriers, and the right-hand side comprises the net positive charge due to vacancy defects in the charge states *q* = +1 and +2. In CeO_2_, the hole contributions are expected to be small but could become non-negligible at high temperatures (see below). In research utilizing first-principles supercell calculations, it is customary to express both defect formation energies and free electron populations as an explicit function of the Fermi level *E*_F_.^49,50^ In contrast, in traditional solid-state chemistry,^29,51^ the charge balance is expressed by the law of mass action, where the Fermi level dependence is more implicit. The two approaches are equivalent, as recently discussed by Anand et al.^31^ The present work utilizes defect equilibrium simulations based on numerical solutions for the self-consistent Fermi level.^23,52^ The simulation code and data are available in the associated content.

For CeO_2_, the *V*_O_ defect formation energies were calculated at different levels of density functional theory (DFT)-based total energy functionals, i.e., GGA,^53^ SCAN,^54^ and HSE,^55^ additionally using the DFT + *U* approach^56^ for GGA and SCAN. However, the GGA + *U* and SCAN + *U* functionals show a spurious delocalization due to the pinning of the *V*_O_ defect state at the CBM for the *U* parameters used here (see Section 4). This result is likely a consequence of the residual delocalization error^57^ in these functionals, precluding the calculation of the proper f^1^ configuration of Ce(3+) states in CeO_2_. In contrast, the HSE hybrid functional calculation yields the expected neutral *V*_O_^0^ defect with two Ce(3+) neighbors (defect bound polarons), where the antiferromagnetic spin alignment is marginally favorable by 1 meV. These findings highlight the need for band gap corrected functionals in systems with charge transition levels in proximity to the band edges. The formation energy of the neutral *V*_O_^0^ is obtained in HSE as Δ*H*_D_^ref^ = 4.00 eV using the fitted elemental reference energy (FERE) for μ_O_^ref^.^58^ Previous DFT + *U* and HSE studies have identified certain vacancy configurations with the polarons in the second coordination shell, slightly lowering the formation energy, by less than 0.1 eV compared to the configuration calculated here.^59−61^ However, for the purpose of the present work, such energy differences are inconsequential. The band gap energy is calculated at *E*_g_ = 3.61 eV in HSE with a dielectric constant of ϵ_r_ = 25.6 including both electronic and ionic contributions. Applying the finite-size corrections of ref (49), the first and second ionization levels of *V*_O_ are obtained in HSE at 0.50 and 0.59 eV below the CBM. The formation of a free polaron through an electron self-trapping mechanism is modeled by a charge transition level at 0.32 eV below the CBM, from a neutral Ce(4+) cation to a negatively charged Ce(3+)^−1^ state at Ce sites in an otherwise defect-free environment. The reduction of the oxidation state increases the Ce–O bond distance from 2.33 to 2.41 Å. Overall, these results are in good quantitative agreement with a previous literature study of CeO_2_ using the HSE functional,^60,62^ and reasonable, more qualitative agreement exists with works using other DFT + *U* or hybrid functionals.^6,22,63−65^ Additionally, the self-trapping of a hole carrier was considered and found to occur at 0.20 eV above the valence band maximum (VBM). Interestingly, the lowest energy configuration found here is an unoccupied state shared between two O sites, akin to the *V*_K_ center in halides,^66^ with a reduced O–O distance of 2.33 Å in the self-trapped polaron state, compared to 2.70 Å in the bulk. Figure S1 in the Supporting Information illustrates the defect-bound and free polarons by showing isosurface plots of the respective spin-densities.

**Figure 3:**
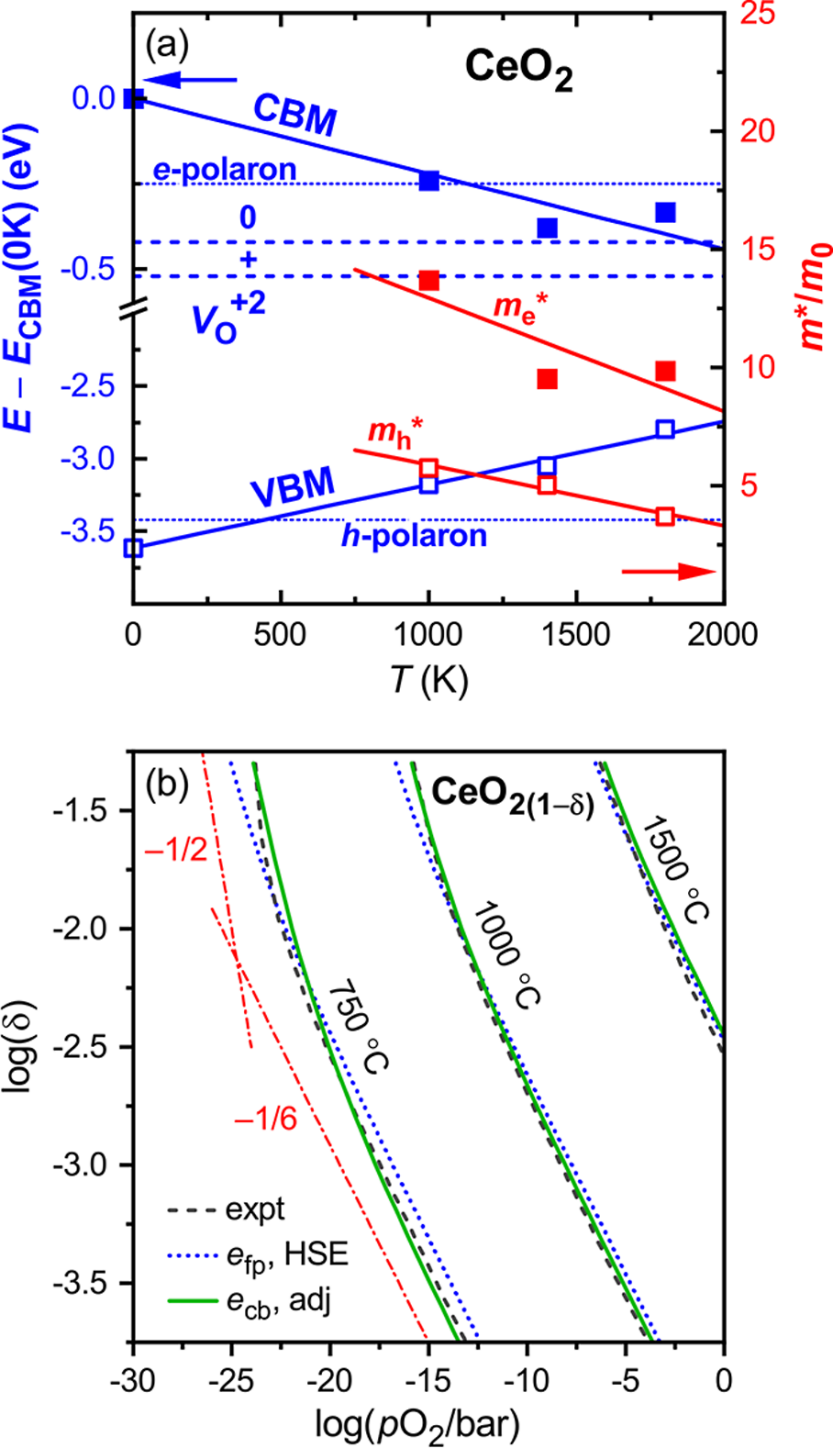
(a) Computational defect model for CeO_2_, showing the relative positions of the *V*_O_ and free-polaron charge transition levels as calculated with the HSE hybrid functional (blue dashed and dotted, left axis). The temperature dependence of the band edges (CBM and VBM, blue solid) and that of the DOS effective masses for electrons and holes (red, right axis) was determined from MD simulations at the GGA + *U* level. Graph shows the adjusted (“adj”) positions of the transition levels with respect to the CBM at 0 K. (b) Brouwer diagram for the present CeO_2(1−δ)_ charged defect models. Experimental data represented by the phenomenological model of ref (45) is shown for comparison (“expt”, gray). Theoretical slopes −1/2 and −1/6 for the neutral and +2 charged O vacancy defect mechanisms, respectively, are indicated (red). The direct first-principles data including localized Ce(3+) free-polaron electron carriers (“e_fp_, HSE”, blue) yields the correct slope at low concentrations but does not describe the change of behavior at high concentrations and lower temperatures. The model using only delocalized conduction band electrons together with slightly adjusted defect formation energies and transition levels (“e_cb_, adj”, green) provides a near-perfect description of experimental observations.

The final ingredient needed to proceed with defect equilibrium calculations for charged defects concerns the temperature dependence of the electronic structure of CeO_2_. The concentrations of band-electrons and band-holes, *n*(e_cb_) and *n*(h_vb_) in eq 11, are obtained from integration of the conduction and valence band density of states (DOS) *D*(*E*), weighted with the Fermi–Dirac distribution. For simplicity, *D*_cb_(*E*) and *D*_vb_(*E*) are approximated with a DOS effective mass model.^67^ As temperature increases, the DOS of the cb and vb continuum states broadens, leading to a reduction of the band gap, accompanied by a reduction of the effective masses *m*_e_* and *m*_h_*. To quantify the temperature dependencies, molecular dynamics (MD) calculations at the GGA + *U* level were performed at 1000, 1400, and 1800 K. Fitting of the electronic structures with linear relationships yields a rate of −2.2 × 10^–4^ eV/K and of +4.3 × 10^–4^ eV/K for the CBM and VBM energies, respectively. The resulting temperature dependence of the band gap of −6.5 × 10^–4^ eV/K is similar to that observed in ZnO,^68^ one of the relatively few cases where the *E*_g_(*T*) dependence is well established. The electron and hole masses are described by *m*_e_^*^(*T*) = (17.8 – 4.8×10^–3^*T*) *m*_0_ and *m*_h_^*^(*T*) = (8.4 – 2.6×10^–3^*T*) *m*_0_, where *T* is in K and *m*_0_ is the electron rest mass. Further details are given in Section 4. Using the 0 K band gap energy from HSE, the resulting temperature dependence of the band edges and effective masses is shown in Figure 3a. In contrast to the band edges, the localized defect and self-trapping levels are expected to be much less affected by temperature^69^ and are here assumed to be constant. As a consequence, the polaron transitions intersect the band edge energies at elevated temperatures (see Figure 3a). In fact, it can be expected that the polaron and band conduction mechanisms become indistinguishable at high temperatures when the amplitude of thermal oscillations becomes comparable to the local distortions that stabilize the polaron.

Figure 3b shows the Brouwer diagram for CeO_2_ for three representative temperatures, comparing thermodynamic simulations based on the present first-principles calculations against experiments, as represented by the model of ref (45). The first computational model to consider is the direct implementation of the above-described results (blue line in Figure 3b), including the HSE calculated band gap, defect formation energy, charge transition levels, and self-trapping energy of the electron-polaron, as well as the temperature dependence of the band edges and effective masses from the MD calculations. Both band- and polaron-carriers are included in this model, according to eq 11. As expected, the data follows largely the −1/6 slope that is characteristic of the charged O vacancy mechanism.^31^ However, it shows subtle discrepancies compared to the experimental reference. First, it does not fully describe the change of slope at high defect concentration, especially at the lower temperature of 750 °C, which likely indicates a transition toward a neutral defect mechanism with a characteristic slope of −1/2. Second, it underestimates the dependence of *p*O_2_ on *T* at constant δ, even in the dilute regime. The latter issue is a manifestation of the underestimated entropy in the polaron model mentioned above in Section 2.1 (17.9 vs 20.5 *k*_B_ measured in ref (29)). The former, more critical issue indicates that the defects mostly remain in the fully ionized *V*_O_^+2^ charge state even at high concentrations. This is a result of the large negative contribution *n*(e_fp_) in eq 11 from the electron polarons, keeping the Fermi level below the defect transition levels (cf. Figure 3a).

To fully reconcile the defect model with the experimental reference data, the following adjustments are made (green line in Figure 3b): first, minor adjustments are made to the defect formation energy Δ*H*_D_^ref^ = 3.83 eV (−0.17 eV adjustment, neutral state) and to the defect levels, 0.42 and 0.52 eV below the CBM at 0 K for the (+1/0) and the (+2/+1) transition, respectively (0.08 eV adjustment closer to the CBM). These small energy adjustments are included in Figure 3a and likely reflect uncertainties associated with the total energy functional. Second, the formation of electron polarons is excluded, resulting in a smaller negative charge from band-electrons only, and allowing for the Fermi level to rise sufficiently to form singly charged and neutral O vacancies. The inclusion of polaron formation clearly deteriorates the agreement with the reference data at low *T* and high defect concentrations. It is likely that the direct HSE-based model overestimates the contribution of the polarons to the charge balance condition (eq 11) because it does not account for the destabilizing interactions between the electron-polarons.^22^

On the other hand, the hole-polarons do not play a significant role for the thermochemical reduction because the self-trapping level lies several eV below the defect state (Figure 3a). However, the VBM energy increases at a sufficiently large rate to cause a significant contribution of band-holes to the defect equilibrium at high temperatures and lower concentrations (see below). Overall, the very close reproduction of the reference data (Figure 3b) validates the adjusted model as a good representation of the real material behavior, notwithstanding the fact that there are numerous open research questions to be addressed by computations for ceria reduction, including short-range ordering of defects and polarons, vibrational free energies, electronic structure, as well as the respective detailed temperature dependencies. It is notable that the present level of agreement is achieved without including defect interactions in the model, as in SCM14 for the case of neutral defects. Even though one could expect stronger repulsive interactions between the charged *V*_O_ defects from electrostatic considerations, it could be possible that the electronic counter-charges generate additional screening effects that minimize the defect interactions. This adjusted CeO_2_ model is used above in Section 2.1 (Figure 2 and Table 1) and will be used below for the analysis of the temperature dependence of the reduction enthalpy and entropy, as well as the simulation of the STCH water splitting process.

### 2.4 Temperature and Composition Dependence of Enthalpy and Entropy

**Figure 4:**
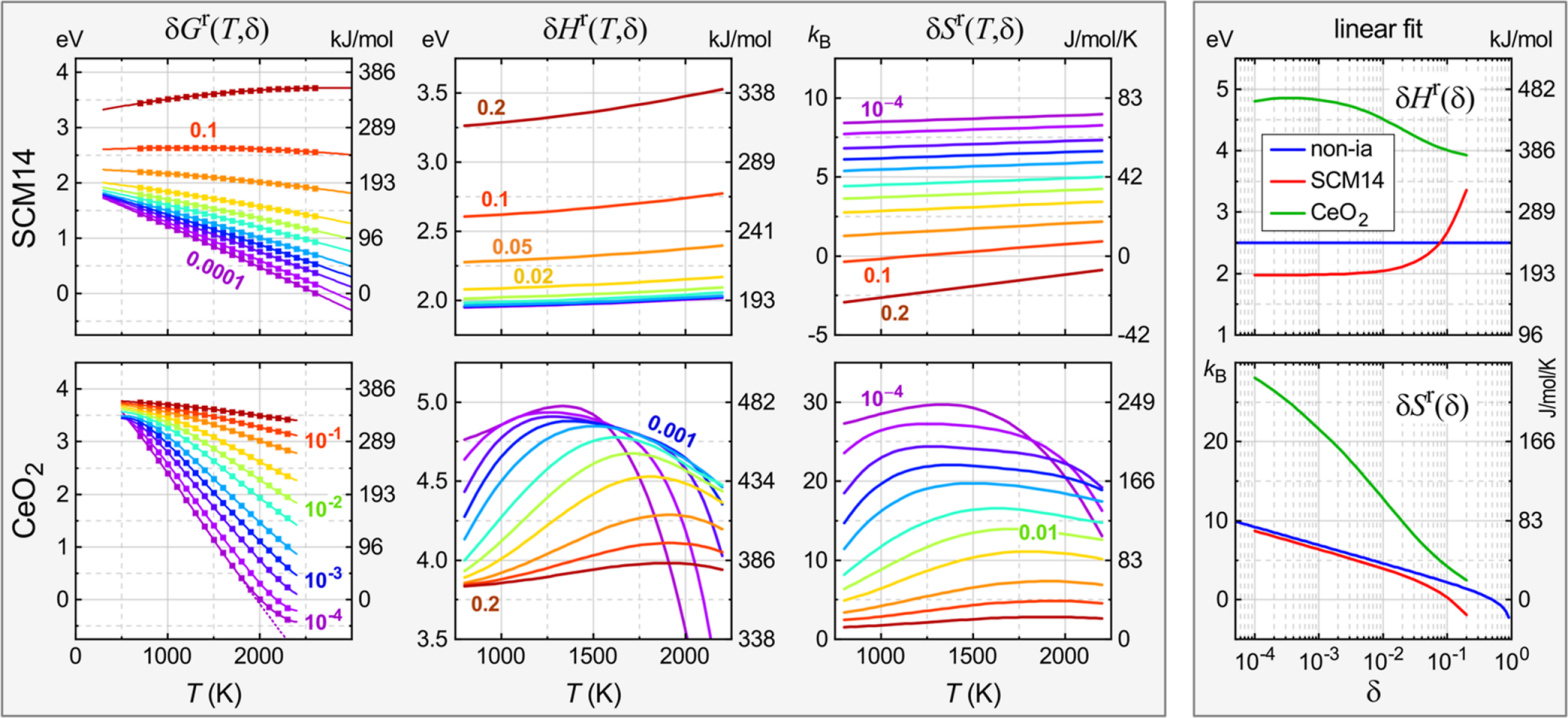
Left panel: temperature and composition dependence of the solid-state free energy of reduction δ*G*^r^(*T*, δ) and the corresponding enthalpy δ*H*^r^(*T*, δ) and entropy δ*S*^r^(*T*, δ) contributions for the SCM14 and CeO_2_ model systems. Results are obtained with a 2nd and 5th order polynomial in eq 8 for SCM14 and CeO_2_, respectively, fitted over the temperature range of the shown data points. Defect concentration is varied logarithmically between 10^–4^ ≤ δ ≤ 0.2 in steps of 1, 2, and 5 units within each decade. Dashed purple line in δ*G*^r^(*T*, δ) of CeO_2_ for the lowest concentration omits the contribution of hole carriers. Right panel: composition dependence of the *T*-averaged enthalpy and entropy contributions from a linear fit of the SCM14 (red) and CeO_2_ (green) models over the interval 1000 K ≤ *T* ≤ 2000 K. In the noninteracting defects case (blue), both δ*H*^r^(δ) and δ*S*^r^(δ) are temperature-independent.

TGA experiments can provide the composition (δ) dependence of the reduction enthalpies and entropies, but within the traditional van’t Hoff method, the solid-state and gas-phase contributions are not separated, and the temperature dependence is averaged over the fitting interval. In contrast, the chemical potential method described above in Section 2.1 provides access to the temperature dependencies δ*H*^r^(*T*) and δ*S*^r^(*T*), by fitting the same underlying data with nonlinear expressions, e.g., a polynomial expansion (eq 9). Figure 4 demonstrates this analysis by way of the model data for SCM14 and CeO_2_ from Section 2.3, and the following discussion is intended to illustrate the additional insights that can be derived from the chemical potential method. For SCM14, the reduction free energy δ*G*^r^(*T*) = −Δμ_O_(*T*) is fitted with a second order polynomial, matching the order of the expansion used for the free energy of defect interaction.^27^ For CeO_2_, where the low and high temperature limits have opposite curvatures with an approximately linear segment in between, a good description is achieved with a fifth order polynomial (cf. Figure 2a).

In the case of the interacting defects in SCM14, Figure 4 shows that both enthalpy and entropy increase with temperature. This observation reflects the fact that the repulsive interactions induce a nonrandom ensemble statistic of the defect distribution.^27^ At lower temperatures, the distribution favors the energetically most favorable configurations, but with increasing *T*, higher energy configurations are excited, causing an increase of the enthalpy δ*H*^r^. The entropy δ*S*^r^ is suppressed by the defect interactions at lower *T*, due to the narrowing of the configuration space, but it increases with *T* as it approaches the ultimate infinite-temperature limit of the random distribution. Thus, the signature of repulsive defect–defect interactions is the gradual increase of both enthalpy and entropy with temperature, while this effect becomes increasingly pronounced at higher defect concentrations.

The CeO_2_ data exhibits a very different behavior, showing nonmonotonic temperature dependencies. After an initial increase, the enthalpy and entropy peak and eventually decay at high temperatures. The relevant processes include the ionization of *V*_O_ defects, which is strongly concentration-dependent, as well as the temperature dependence of the electronic structure (cf. Figure 3a). At the lower end of the concentration range considered in Figure 4, δ = 10^–4^, most vacancies are already ionized at 1000 K, and the behavior of the entropy reflects mostly the temperature dependence of the Fermi level, considering that doubly charged O vacancies add an electronic contribution  to the entropy.^23^ In this situation, the *T* dependence of the CBM provides an additional contribution to δ*S*^r,el^, which is a possible explanation for the experimentally observed entropies in excess of the prediction of the polaron model,^29^ although vibrational entropies could also play a role.^70^ The present defect model yields peak δ*S*^r^ values at 22 and 30 *k*_B_ at δ = 10^–3^ and 10^–4^, respectively (Figure 4), compared to 18 and 25 *k*_B_ in the polaron model (cf. Supporting Information). The downturn of the entropy and, consequently, the enthalpy, at high temperatures is the result of the band gap narrowing (Figure 3a). Above about *T* > 1500 K, the increase of the VBM energy leads to a significant excitation of hole carriers, whose positive contribution to the charge balance condition (eq 11) slows the downward trend of *E*_F_(*T*), thereby reducing δ*S*^r,el^. For illustration, the dashed purple line in Figure 4 shows δ*G*^r^(*T*) for δ = 10^–4^ calculated with only electron contributions to the charge balance. The comparison between this electron-only and the full charge balance simulation proves that the positive curvature of δ*G*^r^(*T*) at high *T*, corresponding to a negative temperature dependence of the entropy, is indeed the result of hole-carrier excitation.

As the defect concentrations increase into the intermediate range 5 × 10^–4^ ≤ δ ≤ 0.01, the Fermi level rises to balance the larger positive defect charge, thereby diminishing the effects of hole-carrier excitation. Instead, we observe a steep increase of δ*S*^r^(*T*) and δ*H*^r^(*T*) around 1000 K in this range, originating from the temperature-activated defect ionization and the associated electronic entropy contribution. For example, at δ = 0.01, the fraction of fully ionized vacancies increases from 22 to 86% between 1000 and 2000 K. However, the ionization process becomes more sluggish at high defect concentrations, with a ratio of only 36% fully ionized defects at 2000 K for δ = 0.1, corresponding to a much more gradual and modest entropy gain.

The temperature-dependent defect ionization has also a revealing consequence for the reduction enthalpy. As seen in the CeO_2_ data in Figure 4 at δ ≈ 0.01, δ*H*^r^ increases from values around 4 eV below 1000 K, close to the neutral vacancy formation energy Δ*H*_D_^ref^ = 3.83 eV in the underlying model, up to almost 5 eV around 1500 K, close to the higher experimental values at low δ that were associated with the upper end of the 2–5 eV target range of defect formation energies for STCH. Thus, the analysis of δ*H*^r^(*T*) helps us to resolve the question from Section 2.2 above of how to relate the observed reduction enthalpies to defect formation energies. At sufficiently high concentrations and low temperatures (but assuming that a defect equilibrium can be established), the vacancies are in the neutral ground state. In this situation, δ*H*^r^ directly reflects the defect formation energy, as in the case of the noninteracting and SCM14 model systems. However, at higher temperatures and lower concentrations, when the defects become fully ionized, the neutral vacancy energy does not influence the defect equilibrium anymore. Instead, δ*H*^r^ now reflects approximately the Fermi-level-dependent formation energy of the charged defects, extrapolated to the CBM at 0 K (an additional small contribution arises due to the nonlinearity in *E*_F_(*T*) caused by the temperature dependence of the effective DOS^23^). This energy is found by adding the ionization energies to the defect formation energy, which yields 3.83 + 0.52 + 0.42 = 4.77 eV within the present CeO_2_ model, close to the value of 4.83 eV at the lower concentration in Table 1. While the localized Ce(3+) polarons were excluded in this model, a similar conclusion holds nevertheless for the case of vacancy-polaron dissociation, except that in this case the ionization energies are measured with respect to the polaron level instead of the CBM, as in the SrMnO_3_ example discussed in Section 2.3, where, however, ionization occurs only at very low concentrations or high temperatures. To recapitulate, the charged defect mechanism can result in apparent reduction enthalpies δ*H*^r^ considerably exceeding the neutral defect formation energy, and the analysis of δ*H*^r^(*T*) and δ*S*^r^(*T*) can reveal important information about the defect ionization process and even the temperature dependence of the electronic structure of the host material.

Before concluding this section, it is useful to consider the *T*-averaged composition dependence of δ*H*^r^(δ) and δ*S*^r^(δ), shown in the right panel of Figure 4, as obtained from a linear fit of δ*G*^r^(*T*, δ) within the interval 1000 K ≤ *T* ≤ 2000 K, similar to the analysis for the data shown in Table 1. Apart from the fact that this analysis excludes the gas-phase contributions, it is analogous to similar plots based on experimental data,^9,10,16,29^ and serves to illustrate the differences in composition dependence between the defect mechanisms, i.e., the noninteracting defects model, the interacting defects in SCM14, and the charged defect mechanism of CeO_2_. In the noninteracting neutral defect case, the enthalpy is composition-independent and the configurational entropy is determined by the statistics of random defect distributions. The interacting defects mostly retain the ideal solution entropy in the low concentration regime but exhibit a significant shortfall in δ*S*^r^(δ) above about δ > 0.01, accompanied by a steep increase in δ*H*^r^(δ). Conversely, the charged defect model of CeO_2_ exhibits a dramatically increased entropy in the low concentration regime, where most defects are fully ionized, accompanied by an elevated enthalpy considerably larger than the neutral defect formation energy. With increasing defect density and the associated decline in the ionization ratio, the entropy deteriorates and approaches the ideal solution limit, while δ*H*^r^(δ) declines toward Δ*H*_D_^ref^ of the neutral vacancy.

In view of the preceding discussion of the temperature-dependent ionization process, it is interesting to revisit the widespread assumption that the main shortcoming of CeO_2_ is the too large O defect formation energy, limiting the degree of reduction under feasible STCH conditions. In fact, the observations deduced from the chemical potential analysis of the CeO_2_ simulations suggest that the defect ionization energies could also play a pivotal role in the STCH process. This hypothesis will be examined quantitatively in the following section. In this context, it is also worth noting that most computational works have so far been restricted to neutral vacancy models,^11,12,14,21^ especially in high-throughput and material screening efforts. Thus, there exist important opportunities for discovery studies that incorporate quantitative prediction of ionization energies.

### 2.5 Simulation of the STCH Water Splitting Cycle

This section analyzes the full gas-splitting cycle within the computational models introduced above, including predictions for hypothetical systems based on a variation of model parameters of CeO_2_. For conciseness, the following discussion addresses the oxidation step only for H_2_O splitting, but an analogous analysis can be made for CO_2_ reduction. Gaseous hydrogen is generated by the reoxidation of the reduced oxide in the presence of H_2_O steam
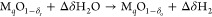
12where Δδ = δ_r_ – δ_o_ is the difference between the O deficiency after reduction (eq 1) and oxidation (eq 12). Thus, the STCH capacity, i.e., the theoretical amount of hydrogen produced upon complete oxidation back to the stoichiometric oxide, is equal to the O deficiency δ_r_ after reduction. On the other hand, the hydrogen yield, which equals Δδ per normalized formula unit, depends on the partial pressure *p*H_2_ in the steam because the gas phase equilibrium
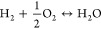
13makes the hydrogen and oxygen partial pressures and corresponding chemical potentials mutually dependent. Using the rigid-rotor and ideal gas law (eq 4) approximations with *H*^°^* = 8.5(9.9) kJ/mol, *S*^°^* = 130.7(188.8) J/mol/K, and *c*_p_ = 3.5(4.0) *k*_B_ for H_2_ (H_2_O), as well as the formation enthalpy of H_2_O(g), Δ*H*_f_ = −2.476 eV (thermochemical data from ref (34)), we can express the O chemical potential, 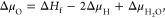
as a function of *T* and the partial pressures *p*H_2_O and *p*H_2_. According to the law of mass action, Δμ_O_ decreases with increasing *p*H_2_, i.e., with increasing hydrogen-to-steam ratio, causing an increase in δ_o_ and diminishing the STCH yield. While this general trend is clearly observed experimentally,^7,8^ there is currently no quantitative understanding on how this trade-off is affected by the defect properties, and on where the theoretical limits are for the yield as a function of *p*H_2_.

**Figure 5:**
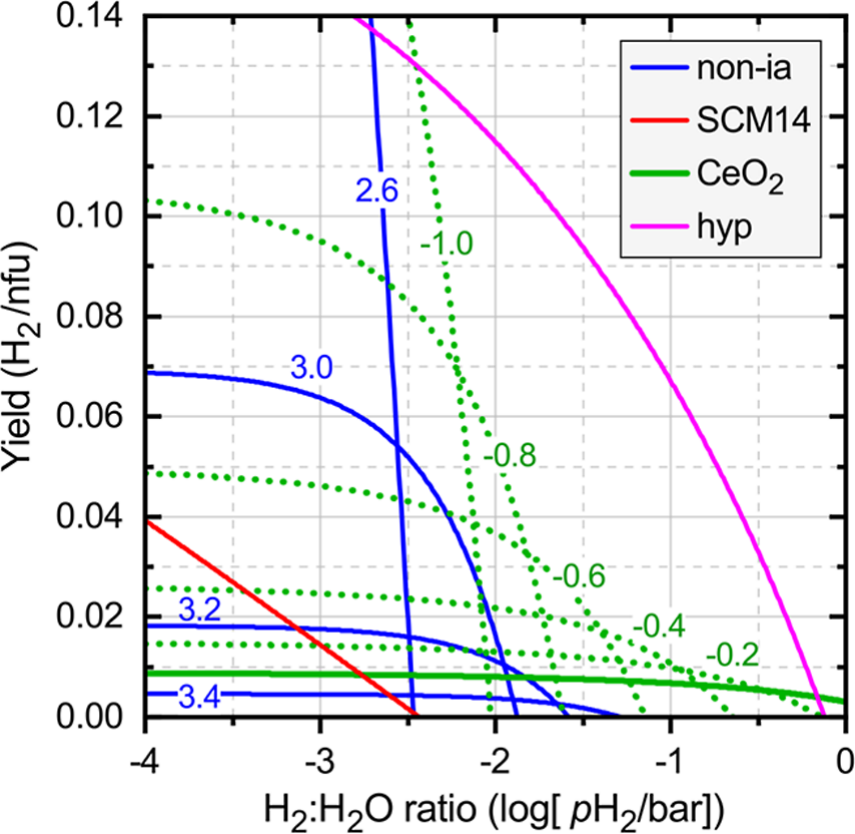
Simulation of the thermochemical H_2_O splitting cycle with reduction at *T*_r_ = 1400 °C and *p*O_2_ = 10^–4^ bar and with oxidation (gas-splitting) at *T*_o_ = 850 °C and *p*H_2_O = 1 bar. The figure shows the H_2_ yield per normalized formula unit (nfu, cf. eq 1), as a function of *p*H_2_ for the following cases: (blue) noninteracting O vacancies for a range of different defect formation energies Δ*H*_D_^ref^, indicated in units of eV. (red) Interacting defect model for SCM14. (green) Charged defect model for CeO_2(1−δ)_ (solid line) and for modified CeO_2_-like systems (dotted lines), where Δ*H*_D_^ref^ of the neutral vacancy is reduced by the numbers indicated. (magenta) Model for a hypothetical (hyp) system with additional modifications of charge transition levels and CBM temperature dependence.

Comparing the different defect mechanisms, Figure 5 plots the H_2_ yield as a function of *p*H_2_, where the reduction condition is taken as *T*_r_ = 1400 °C at *p*O_2_ = 10^–4^ bar, with oxidation (water splitting) at *T*_o_ = 850 °C and *p*H_2_O = 1 bar. Within the scenario of ideal (noninteracting) charge-neutral defects, we observe that significant H_2_ yields above 2% occur only under relatively dilute H_2_/H_2_O ratios below *p*H_2_ = 10^–2^ bar. For sufficiently low defect formation energies and H_2_ partial pressures, the model predicts high yields above 10% and more. However, in realistic systems like SCM14, defect interactions suppress the differential reduction entropy δ*S*^r^, especially at high defect concentrations (see Section 2.4). Since δ*S*^r^ equals the temperature derivative of the O chemical potential Δμ_O_ (see Section 2.1 and ref (23)), a reduced entropy implies an increased O chemical potential at the lower oxidation temperature. However, according to the gas-phase equilibrium (eq 13), the higher Δμ_O_ necessitates a lower Δμ_H_ and corresponding H_2_ partial pressure. These unfavorable implications of defect interactions are clearly illustrated by Figure 5, where SCM14 underperforms compared to the noninteracting defect model either in yield or in *p*H_2_, depending on the defect formation energy used for the non-ia model. Similar limitations in the H_2_/H_2_O ratio exist also for other complex oxide systems, such as BaCe_0.25_Mn_0.75_O_3(1−δ)_ (BCM) or Sr–La–Mn–Al oxides.^7,8^

Given the much larger reduction entropy associated with the charged defect mechanism, CeO_2_ is able to spilt water at much more concentrated H_2_/H_2_O ratios. This outstanding behavior is experimentally well established^71^ and follows from the phenomenological thermodynamic model.^8,45^ The present charged defect model allows to explore hypothetical scenarios of the water splitting behavior upon modification of the defect properties. Considering the high reduction enthalpy and low capacity of ceria, the most immediate design strategy might appear to be a lowering of the O vacancy formation energy. Figure 5 shows the H_2_ yield for the present CeO_2_ model together with the results obtained when lowering Δ*H*_D_^ref^ in increments of 0.2 eV while keeping all other modeling parameters fixed. The CeO_2_ capacity of just about 1% increases considerably, reaching 10% for a reduction of −0.8 eV. However, the ability of ceria to split water at high H_2_/H_2_O ratios is severely impacted at the same time, and *p*H_2_ drops below 10^–2^ bar for yields above 5%. Even though the “modified CeO_2_” model significantly outperforms the noninteracting neutral defect model, the increased capacity largely negates the unique advantage of high H_2_/H_2_O ratios.

If the benefit from a simple reduction of defect formation energy is limited, it is important to address the question of what would be the theoretical limits of the charged defect model for a hypothetical material with advantageous but still realistic properties. To this end, the possibility of smaller defect ionization energies and a stronger CBM temperature dependence is considered, both of which would be beneficial for better water splitting performance.^23^ For example, the CBM could decrease about twice as fast as in CeO_2_ at a rate of −5 × 10^–4^ eV/K, with a band gap large enough to prevent the excitation of hole carriers. While larger electron masses would be beneficial, a stronger temperature dependence of the CBM would be more likely to occur in a system with a smaller electron mass, so we could assume, for example, *m*_e_^*^(*T*) = (10 – 2×10^–3^*T*) *m*_0_. Further, while O vacancies are quite generally expected to introduce localized defect levels in transition metal oxides, the charge transition levels could fall close to the CBM in the case of a favorable electronic configuration^67^ and strong dielectric screening. For the hypothetical, we will assume 0.1 and 0.2 eV for the first and second ionization energies, respectively. Finally, selecting the defect formation energy Δ*H*_D_^ref^ = 3.6 eV such to maximize the H_2_ yield at *p*H_2_ = 0.1 bar, the magenta line in Figure 5 shows the water splitting performance for this hypothetical oxide. It eclipses the other defect models by a considerable margin, including the CeO_2_ model with reduced defect formation energies. The predicted H_2_ yield is as high as 7% at a highly concentrated H_2_/H_2_O ratio of about 1:10, for the same reduction and oxidation conditions described above. Clearly, the discovery of such a material would be a great leap forward toward a commercially viable STCH technology.

## 3 Conclusions

The van’t Hoff method essentially amounts to an Arrhenius transformation of *p*(*T*) partial pressure vs temperature data but suffers from a residual non-Arrhenius term originating from gas-phase contributions, leading to a deviation from the ideal linear relationship even when the solid-state enthalpy and entropy are constant. While this problem can be mitigated by subtracting the gas-phase term, the bigger shortcoming still remains, which is that the transformation is not suitable for more general situations with temperature-dependent enthalpies and entropies. The present work uses simulated *p*O_2_(*T*) data for oxide reduction in different defect models to demonstrate the alternative “chemical potential method”, utilizing the functional −Δμ(*T*) relationship instead. For the cases of constant enthalpy and entropy, the analysis is similarly simple as in the van’t Hoff method, with δ*S* being obtained from the slope and δ*H* from the 0 K intercept. However, the real benefit of this approach is the access to the temperature dependence of the entropy δ*S*(*T*) as the *T*-derivative, which can be readily obtained after fitting the data with suitable functional forms. Using polynomial fits, the temperature-dependent reduction enthalpies and entropies were determined for first-principles-derived defect models for interacting (SCM14) and charged (CeO_2_) oxygen vacancy defects. This analysis illustrates how the *T* dependence of δ*H*^r^ and δ*S*^r^ reveals important information about the respective defect mechanisms, including (detrimental) defect interactions and (beneficial) defect ionization.

The charged defect mechanism is a promising direction for the design and discovery of oxides for solar thermochemical hydrogen generation because the additional electronic entropy contribution counteracts the dilemma that high H_2_ yields necessitate dilute hydrogen-to-steam ratios in most oxides. Ceria is currently the only oxide where a charged defect mechanism has been unambiguously confirmed under STCH relevant conditions. The analysis of the model data suggests that the unique behavior of CeO_2_ originates from the relatively small ionization energies. The ionization can occur either through the dissociation of localized electron polarons from the defect or by excitation into the delocalized continuum states of the conduction band. Both scenarios yield similar electronic entropy contributions, can coexist, and become essentially indistinguishable at high temperatures where thermal vibrations become comparable to the lattice distortions associated with polaron self-trapping. Within the charged defect regime, the reduction enthalpy corresponds approximately to the sum of the defect formation and ionization energies and therefore should not be associated with the formation energy of the neutral defect alone. Even with its relatively small ionization energies, CeO_2_ transitions from the charged to the neutral mechanism at higher defect concentrations and lower temperatures, explaining the large variations of δ*H*^r^(δ, *T*) and δ*S*^r^(δ, *T*). If the ionization energies are substantially larger, as illustrated for the example of cubic SrMnO_3_, the defects remain in the neutral state throughout the range of STCH relevant concentrations and temperatures, thereby precluding the electronic entropy gain. This situation seems to be present for many STCH oxides containing transition metals with localized 3d electrons, where the H_2_ yield quickly deteriorates with increasing H_2_/H_2_O ratio.

The development of high-performing STCH oxides will greatly benefit from an improved understanding of the ionized vs neutral defect mechanism across a broad spectrum of materials. To guide expectations about theoretical limits for STCH water splitting and to elucidate signatures of promising material behavior, hypothetical systems were considered, which embody a variation of the CeO_2_ model parameters. This analysis revealed that a lowering of the defect formation energy of CeO_2_ alone would provide a significant but somewhat limited improvement. However, a much more pronounced benefit would result from a material with smaller ionization energies and stronger *T* dependence of the conduction band energy. Thus, whereas previous material discovery studies almost exclusively focused on the charge-neutral defect mechanism, important opportunities await in the realm of charged defects, where electrons are more easily dissociated or excited into the band continuum.

## 4 Methods

The *V*_O_ defect formation energies in CeO_2_ were calculated in GGA,^53^ SCAN,^54^ and HSE,^55,72^ following the general approach described in ref (58). The DFT + *U* method^56^ was employed for GGA + *U* (*U*_f_ = 2.0 eV, *U*_d_ = 1.5 eV^73^) and SCAN + *U* (*U*_f_ = 1.0 eV, *U*_d_ = 0^27^). Conventional settings were used for the HSE functional, i.e., 25% fock exchange mixing and 0.2 Å^–1^ for the range separation parameter.^55^ These calculations were performed with the projector augmented wave (PAW) method of the VASP code,^74,75^ using 96 atom supercells and a 2 × 2 × 2 *k*-mesh for Brillouin zone sampling without reductions in the exchange integral in the case of the HSE hybrid functional (i.e., the final results were obtained with the VASP setting NKRED = 1). Spin polarization was included for open-shell configurations. Analogous HSE calculations were performed for charged O vacancies and Mn(3+) polarons in SrMnO_3_ using an 80 atom supercell (results are fully consistent with those reported in ref (27)).

Due to the short bond length, accurate calculations of the molecular O_2_ energy require using the hard “O_h” PAW potential with an energy cutoff of 900 eV or higher.^58^ However, in the oxide supercell calculation, this setting would add excessive and unnecessary computational expense. The present calculations utilized the soft “O_s” PAW pseudopotential with a much lower energy cutoff of 380 eV. Appropriate elemental reference energies μ_O_^ref^ for this PAW potential were determined previously in ref (58) for GGA and HSE and in ref (27) for SCAN, by calculating the O_2_ binding energy with “O_h” and adding it to the free atom energy of “O_s”. Alternatively, the FERE,^76,77^ which improve the prediction of thermochemical properties, avoid the calculation of the molecular energy altogether. The unadjusted HSE results shown in Figure 3b were obtained with the respective oxygen FERE for HSE from ref (58).

Vibrational free energy contributions are not considered in the present work. In a previous work on FeAl_2_O_4_,^24^ we found that such contributions are only of minor significance (see also Supporting Information). Furthermore, Grieshammer et al.^70^ calculated vibrational entropies for defects in CeO_2_ and obtained a modest combined value of 2.5 *k*_B_ for one *V*_O_^+2^ defect with two Ce_Ce_^–1^ polarons. However, this work also reported surprising, much larger entropy values for individual charged defects originating mainly from the relaxation volume. While this issue deserves further study, it should be noted that the original total energy expression in the plane-wave pseudopotential formalism^78^ is well-defined only for charge-neutral systems. Consistent total energy expressions for charged defect supercells were obtained by including the energy contribution of the electronic counter-charge (Fermi level) in conjunction with a potential alignment correction that ensures a consistent energy scale between the defected and the defect-free cells.^49,79,80^ However, this approach does not consider volume changes. The original work on charged-cell total energies^79^ also considered the possibility to obtain a well-defined total energy based on compensation by the homogeneous background charge, resulting in an additional energy term that depends on the volume. Thus, the uncorrected charged-cell energy may not be suitable for equation of state calculations.

MD calculations were performed at the GGA + *U* level in a 192 atom CeO_2_ supercell at the Γ point. Using the Nosé algorithm^81^ and a time step of 2 fs, 10 snapshots were taken after successive equilibrations for 2 ps, for a total simulation time of 20 ps. For each snapshot, a static calculation was performed with a 2 × 2 × 2 *k*-mesh, from which the respective DOS effective masses were determined for the corresponding temperature, as described in ref (67). The MD calculations were performed at a constant, 0 K relaxed volume. While including the thermal expansion directly in MD is possible,^82^ this was not attempted here. The present model utilizes the anchoring of the charged defect energy at the average electrostatic potential,^58,83^ which requires the separation of the temperature dependence of the band gap into individual contributions from VBM and CBM. Volume relaxation impairs the common energy scale needed for this separation for reasons related to the previous paragraph, although it could be restored by potential alignment. It is expected that the thermal expansion would cause an additional but smaller contribution to the band gap reduction.

## Data Availability

Data and computer codes are available in a Zenodo repository (DOI: https://doi.org/10.5281/zenodo.11008998). It contains the codes for the thermodynamic simulations, the generated data, the atomic structure geometries of the first-principles defect calculations reported here, as well as two spreadsheets with the rigid-rotor + ideal-gas-law equations, an expression for adding the vibrational free energy in the case of O_2_, data for the fits in Figures 2 and 4, as well as results and analysis of the first-principles calculations (defect energies and MD simulations).

## Supporting Information Available

Additional discussion of vibrational free energy contributions in gas and solid phases, expressions for the ideal solution entropy of Ce(3+) and Mn(3+) free-polarons, and spin-density iso-surface plots of the *V*_O_ defect and electron- and hole-polarons (PDF)
