# Supplementary material for: Chemical Potential Analysis as an Alternative to the van’t Hoff Method: Hypothetical Limits of Solar Thermochemical Hydrogen
Source: J Am Chem Soc. 2024 May 13;146(20):14114–27. doi: 10.1021/jacs.4c02688 (PMC11117408; doi:10.1021/jacs.4c02688)
Supplement: Supplementary file 1 — ja4c02688_si_001.pdf [file ja4c02688_si_001.pdf]

Chemical potential analysis as alternative to the van't Hoff method: Hypothetical limits of solar thermochemical hydrogen

Stephan Lany,[1,*]

[1]National Renewable Energy Laboratory, Golden, CO 80401, USA

April 21, 2024

*Stephan.Lany@nrel.gov

Supporting information

1 Role of vibrational free energies

As mentioned in the main text, the ideal gas expression for the O_2 chemical potential remains accurate up to about 2000 K when using the constant value of $c_\mathrm{p} = 3.5\ k_\mathrm{B}$ from the rigid rotor approximation. At higher temperatures, the excitation of phonons above the zero point energy (ZPE) becomes more significant. The more general form of the gas phase chemical potential (eq. 4, main text) for a temperature dependent heat capacity reads

$$\Delta\mu(p,T) = \left[H^{\circ *} + \int_{T^*}^{T} c_\mathrm{p}(T)dT\right] - T\left[S^{\circ *} + \int_{T^*}^{T} \frac{c_\mathrm{p}(T)}{T}dT\right] + k_\mathrm{B}T\ln(p/p^\circ), \quad \text{(S1)}$$

where one can use the tabulated values of $c_\mathrm{p}(T)$ [1] to calculate $\Delta\mu(p,T)$ with vibrational effects if needed or desired. The repository contains a spreadsheet for calculating $\Delta\mu(p,T)$ within the rigid-rotor approximation with respective values for $H^{\circ *}$, $S^{\circ *}$, and c_p for O_2, H_2, and H_2O. For O_2, it also contains a simple correction formula, so to obtain results matching eq. S1 with data from Ref. [1].

For defect models based on first-principles calculated defect formation energies, the leading free energy contributions are the ideal gas, configurational, and electronic (for charged defects) free energies. Vibrational effects usually play a lesser role that these, but are nevertheless a potentially important finite-temperature effect to consider. The Dulong-Petit law suggests an approximate cancellation of vibrational free energies for reactions involving only solid phases, which often justifies to omit vibrational effects from explicit consideration. If vibrational free energies are excluded in the solid state, the rigid-rotor approximation for $\Delta\mu(p,T)$ suggests itself for consistency. However, since thermochemical reduction of oxides involves both solid and gas phases, a good cancellation is not obvious. Our defect models for $SrMnO_3$ [2] and CeO_2 (present work) provide an excellent description of defect formation over a wide temperature range up to 2000 K without consideration of vibrational effects, suggesting that these contributions are minor. In Ref. [3], we performed phonon calculations for hercynite $FeAl_2O_4$ in the quasi-harmonic approximation

for supercells with and without the O vacancy defect, showing that the free energy contributions to defect formation remained well below 0.1 eV within this temperature window. Thus, while further studies of phonon properties are welcome, there is currently little evidence suggesting that vibrational contributions play a major role in the thermochemical reduction reaction (see also discussion in the methods section of the main text).

2 Ideal solution entropy for electron polarons

To calculate the absolute and differential electronic entropies associated with a polaron mechanism, we consider that each O vacancy creates 2 excess electrons. Therefore, in CeO_2, the fractional concentration of Ce(3+) polarons on the Ce sublattice equals 4δ for a normalized formula unit of reduced ceria, $Ce_{0.5}O_{1-\delta}$. If these polarons distribute randomly, the corresponding absolute and differential electronic-configurational entropies are, respectively,

$$\begin{aligned} \Delta S^{\mathrm{r,pol}} &= -\frac{k_{\mathrm{B}}}{2}[4\delta \ln(4\delta) + (1-4\delta)\ln(1-4\delta)], \\ \delta S^{\mathrm{r,pol}} &= 2k_{\mathrm{B}} \ln\left(\frac{1-4\delta}{4\delta}\right). \end{aligned} \tag{S2}$$

A similar consideration for perovskite $SrMnO_{3(1-\delta)}$ yields

$$\delta S^{\mathrm{r,pol}} = 2k_{\mathrm{B}} \ln\left(\frac{1-6\delta}{6\delta}\right). \tag{S3}$$

At, e.g., $\delta = 0.001$, this contribution amounts to 11.03 and 10.22 k_{B} in CeO_2 and $SrMnO_3$, respectively, which adds to the atomic-configurational (differential) reduction entropy. Note, however, that the large dissociation enthalpy of 1.40 eV per vacancy in $SrMnO_3$ prevents the ionization under conditions relevant for STCH, as discussed in the main text.

3 Defect bound and free polarons

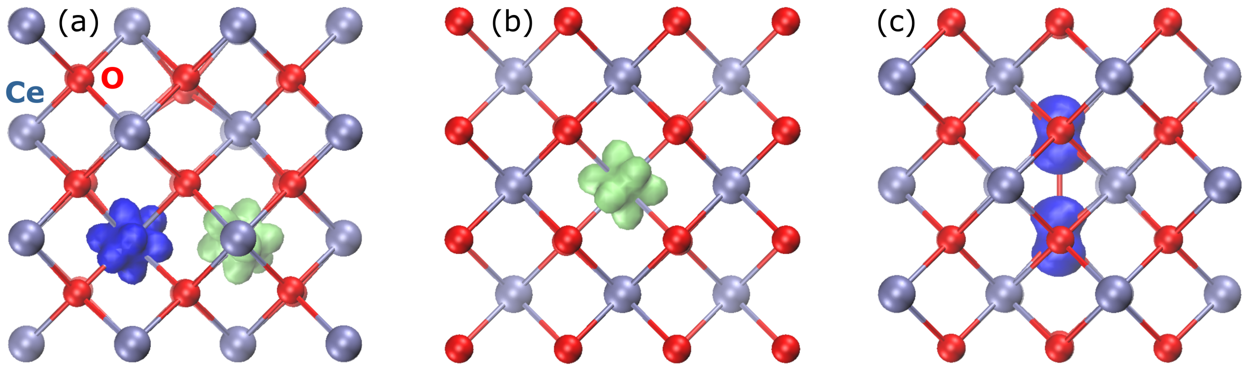

Figure S1: CeO_2 ball-stick atomic structure models and spin-density isosurfaces for (a) the charge-neutral O vacancy with anti-ferro-magnetically aligned Ce(3+) defect-bound polarons, (b) the Ce(3+) free electron-polaron, and (c) the hole-polaron formed in a O-O dimer configuration.

References

[1] NIST-JANAF thermochemical tables. https://janaf.nist.gov/. Accessed: 2024-01-08.

[2] Anuj Goyal, Michael D Sanders, Ryan P O'Hayre, and Stephan Lany. Predicting thermochemical equilibria with interacting defects: $Sr_{1-x}Ce_xMnO_{3-\delta}$ alloys for water splitting. *PRX Energy*, 3(1):013008, 2024.

[3] Samantha L Millican, Jacob M Clary, Charles B Musgrave, and Stephan Lany. Redox defect thermochemistry of $FeAl_2O_4$ hercynite in water splitting from first-principles methods. *Chemistry of Materials*, 34(2):519–528, 2022.
